# The ovipositor cue indole inhibits animal host attraction in *Aedes aegypti* (Diptera: Culicidae) mosquitoes

**DOI:** 10.1186/s13071-022-05545-8

**Published:** 2022-11-12

**Authors:** Amir Dekel, Evyatar Sar-Shalom, Yuri Vainer, Esther Yakir, Jonathan D. Bohbot

**Affiliations:** Department of Entomology, The Robert H. Smith Faculty of Agriculture, Food and Environment, The Hebrew University of Jerusalem, 76100 Rehovot, Israel

**Keywords:** *Aedes aegypti*, Indole, DEET, IR3535, (*R*)-1-octen-3-ol, OR8, Repellent

## Abstract

**Background:**

Mosquitoes are responsible for disease transmission worldwide. They possess the ability to discriminate between different ecological resources, including nectar sources, animal hosts and oviposition sites, a feature mediated by their olfactory system. Insect repellents, such as* N*,*N*-diethyl-meta-toluamide (also called DEET), have been shown to activate and inhibit mosquito odorant receptors, resulting in behavioral modulation. This and other repellents currently available for personal protection against mosquitoes are topically applied to the skin and operate at a short range. In our search for potential long-range inhibitors of attractants to human hosts, we have hypothesized that the shared chemical similarities between indole and DEET may confer the former with the ability to block odorant receptor function and inhibit human host attraction in a similar way as DEET.

**Methods:**

We used the two-electrode voltage clamp system to assay *Xenopus laevis* oocytes as a platform to compare the pharmacological effect of commercially available insect repellents and indole on the *Aedes aegypti* (*R*)-1-octen-3-ol receptor, OR8, a receptor involved in the decision-making of female mosquitoes to identify human hosts. We also conducted arm-in-a-cage and wind-tunnel bioassays to explore the effect of indole on human host-seeking female *Aedes aegypti* mosquitoes.

**Results:**

Our results demonstrate that indole inhibited the *Aedes aegypti* (*R*)-1-octen-3-ol receptor OR8. In our arm-in-a-cage assay, 1 M of DEET reduced mosquito visits on average by 69.3% while the same indole concentration achieved 97.8% inhibition. This effect of indole on flight visits was dose-dependent and disappeared at 1 μM. In the flight tunnel, indole elicited on average 27.5% lower speed, 42.3% lower upwind velocity and 30.4% higher tortuosity compared to the control.

**Conclusions:**

Indole significantly inhibits OR8 activation by (*R*)-1-octen-3-ol, mosquito visits to a human hand and long-range human host-seeking. The volatility of indole may be leveraged to develop a novel insect repellent in the context of personal mosquito protection.

**Graphical abstract:**

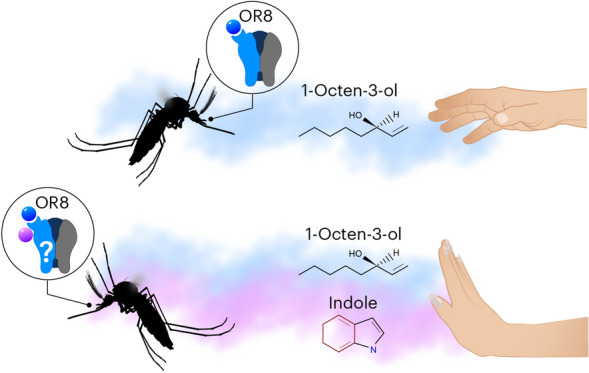

**Supplementary Information:**

The online version contains supplementary material available at 10.1186/s13071-022-05545-8.

## Introduction

Mosquitoes are a major vector of infectious diseases worldwide due to their ability to transmit pathogens and are also a major source of annoyance. A spatial repellent, such as* N*,*N*-diethyl-meta-toluamide (DEET), inhibits mosquito attraction at various distances from the host, ranging from a few centimeters to close to 1 m [1–3]. However, despite the protection such repellents provide, mosquitoes are still able to efficiently locate potential human hosts, hover around them and locate any unprotected area that can be targeted for biting. It is therefore desirable to find alternative solutions with an extended protection range, such as volatile odorant repellents [1]. Plant volatiles, such as geraniol, citral, citronellal, eugenol and anisaldehyde, have been shown to exhibit spatial repellency against mosquitoes [4]. In the future, these plant odorants may play a major role in the development of spatial repellent technology.

Mosquito repellents such as DEET exhibit low volatility and have been reported to act at ranges of between 4 and 8 cm when used in cages [2]. These compounds exhibit complex interactions with odorant receptors (ORs), activating some ORs while inhibiting others [5–7]. Notwithstanding its odor masking properties [8–10], DEET alone activates the *Aedes aegypti* indole-sensitive receptor AaegOR2 and also inhibits the (*R*)-1-octen-3-ol-activated receptor AaegOR8 [5]. The *OR8* gene is expressed in basiconic sensilla on the maxillary palps of mosquitoes belonging to species of the Anopheline and Culicine families [11, 12]. This phylogenetically conserved receptor specifically responds to the (*R*)-1-octen-3-ol enantiomer in the nanomolar range [13, 14] and is believed to synergize the effect of CO_2_ in the attraction to human hosts [15–17].

Indole alone does not mediate oviposition in *Anopheles gambiae* (Diptera: Culicidae) [18] and *Ae. aegypti* [19] unless combined with 3-methyl-1-butanol in *An. gambiae* [18] and nonanal,* p*-cresol, phenol and dimethyltrisulfide in *Culex *spp. (Diptera: Culicidae) [20]. High indole dosages have been demonstrated to repel the oviposition of mosquitoes such as *Toxorhynchites *spp. (Diptera: Culicidae) [21], *Culex quinquefasciatus* [22], *Aedes *spp. [19] and *An. gambiae* [23].

**Fig. 1:**
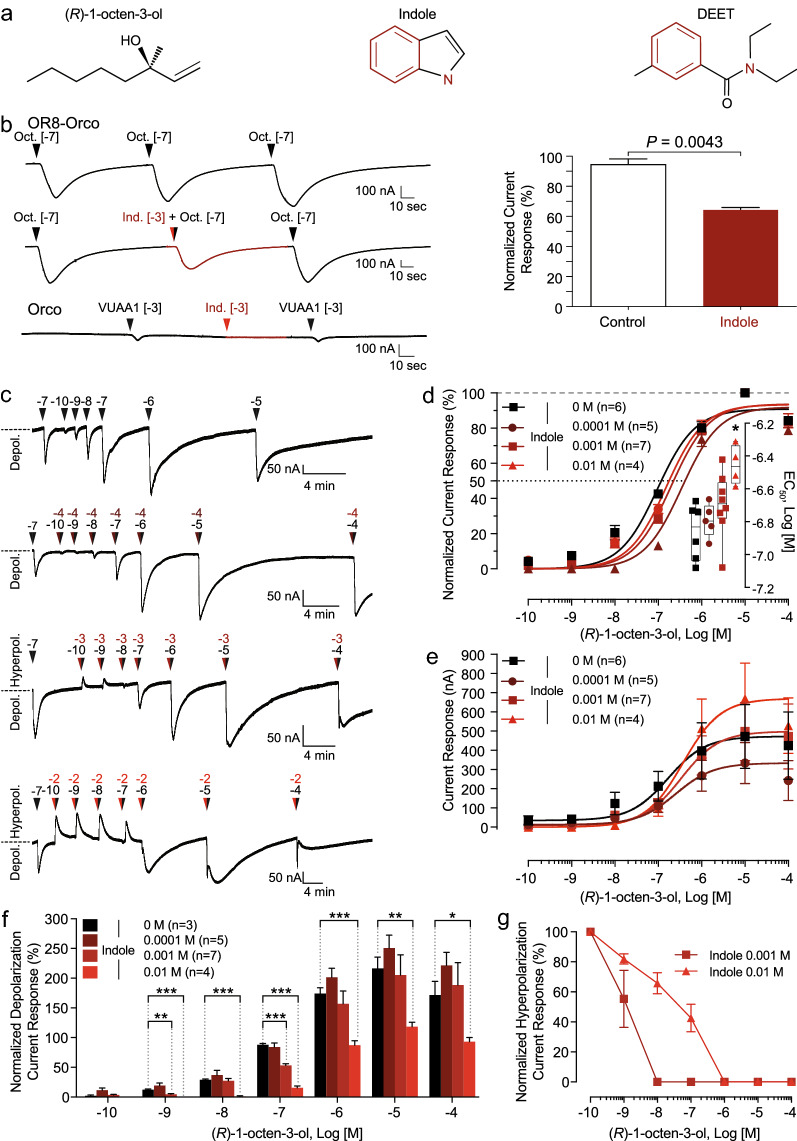
Indole inhibits OR8-Orco activation by (*R*)-1-octen-3-ol. **a** (*R*)-1-octen-3-ol is an alkenyl alcohol and a potent ligand of OR8. Indole is an aromatic bicyclic organic compound. DEET is a benzyl-ester sharing a benzene ring and a nitrogen atom with indole (red features). **b** Representative current traces of AaegOR8-Orco activation by 10^–7^ M (*R*)-1-octen-3-ol (black arrowheads) or by a mixture of 10^–3^ M indole (red arrowhead) and 10^–7^ M (*R*)-1-octen-3-ol (black arrowhead). Normalized responses of AaegOR8-Orco to 10^−7^ M (*R*)-1-octen-3-ol alone (control) or in combination with 10^−3^ M indole (indole). Statistical significance was determined using the Wilcoxon–Mann–Whitney test (*n* = 5–6). Orco-injected oocytes responded to 10^–3^ M of the Orco agonist VUAA1 (positive control) but not to 10^–3^ M indole (*n* = 4). **c** Representative current traces of AaegOR8-Orco activation by (*R*)-1-octen-3-ol alone or in the presence of 10^–4^ M, 10^–3^ M or 10^–2^ M indole. Arrowheads above the traces indicate the onset of the odorant stimulus, black arrowheads represent (*R*)-1-octen-3-ol and red arrowheads represent indole. **d** Concentration–response relationships of AaegOR8-Orco in response to increasing indole concentrations. Current amplitudes were normalized to the maximum response. Statistical significance was determined using a one-way analysis of variance (ANOVA; *P* = 0.0142) followed by a Dunn’s multiple comparisons test (*n* = 4–7). **e** Non-normalized concentration-current response relationships (*n* = 4–7). **f** Histogram of the depolarization current response amplitudes of AaegOR8-Orco in response to (*R*)-1-octen-3-ol alone (black) or in combination with various indole concentrations (shades of red). Statistical significance was determined using the multiple t-test and by the Holm-Sidak method (*P ≤ 0.05; **P ≤ 0.01; ***P ≤ 0.001; *n* = 3–7). **g** Concentration–response relationships of indole-induced hyperpolarization currents exhibited by AaegOR8-Orco-injected oocytes. AaegOR8-Orco,* Aedes aegypti* odorant receptor 8-odorant coreceptor; DEET, *N*,*N*-Diethyl-*meta*-toluamide

Noting that DEET and indole exhibit overlapping chemical features, including the presence of an aromatic ring and a neighboring nitrogen atom (Fig. 1a), we hypothesized that indole might interfere with OR8 function. In the present study we used the two-electrode voltage clamp method to assay *Xenopus laevis* oocytes expressing *Ae. aegypti OR8* and showed that indole inhibited OR8-mediated current depolarizations elicited by (*R*)-1-octen-3-ol. We also showed that sustained exposures to indole and repellents generated an increase of the current baseline. We observed that co-administration of indole and (*R*)-1-octen-3-ol evoked significant hyperpolarizations that were concentration dependent. In light of these pharmacological results, we tested the potential behavioral effect of indole on animal host-seeking female *Aedes aegypti* using an arm-in-a-cage assay, providing evidence that indole acted as a repellent at various doses. Using a wind tunnel, we showed that indole decreased anemotactic flight elicited by a synthetic human host blend composed of CO_2_ and 1-octen-3-ol. Our findings suggest that volatile compounds such as indole may be discovered based on pharmacological knowledge of ORs. The volatility of indole and the phylogenetic conservation of the CO_2_ and octenol receptors in the Culicidae family may be leveraged to develop a novel insect repellent with a broad spectrum of action in the context of personal mosquito protection.

## Methods

### *Aedes aegypti* mosquitoes

The mosquito laboratory-reared colony developed by Prof. Joel Margalit was maintained in an air-controlled insect chamber at 26 °C, 80% relative humidity (RH) and 12:12-h light/dark photoperiod. Mosquito larvae were kept in plastic pans containing 1 l of water. Larvae were fed with a ground mixture of Novocrabs feed (JBL GmbH & Co., Neuhofen, Germany), mice feed and dry yeast until adult emergence. Pupae were transferred into plastic cages. and eclosed adults were allowed to mate and feed ad libitum on a 10% sucrose solution. Females were fed with cow blood using a membrane feeding system.

### AaegOR8-Orco messenger RNA expression in *Xenopus laevis* oocytes

In vitro transcription and two-microelectrode voltage-clamp electrophysiological recordings were carried out as previously described [14]. Both the AaegOR8 OR and the* Ae. aegypti* odorant coreceptor (AaegOrco) were synthesized using the mMESSAGE mMACHINE® SP6 kit (Thermo Fisher Scientific, Waltham, MA, USA) from the linearized pSP64tRFA expression vector. *Xenopus laevis* oocytes were manually collected, separated and treated for 30 min with a 8 mg/ml collagenase solution at 18 °C to remove the follicular layer. Stage V–VI oocytes were rinsed in Ringer solution (96 mM NaCl, 2 mM KCl, 5 mM MgCl_2_ 5 mM HEPES, pH 7.6) and microinjected with a mixture of 1 μl AaegOR2 (3 μg/μl), 1 μl AaegOrco (3 μg/μl) and 1 μl of double-distilled water. Injected oocytes were maintained at 18 °C for 3 days in Ringer’s solution (96 mM NaCl, 2 mM KCl, 5 mM MgCl_2_, 0.8 mM CaCl_2_, 5 mM HEPES, pH 7.6) supplemented with 5% dialyzed horse serum, 50 μg/ml tetracycline, 100 μg/ml streptomycin and 550 μg/ml sodium pyruvate.

Whole-cell currents were monitored and recorded using the two-microelectrode voltage-clamp technique. Holding potential was maintained at − 80 mV using an OC-725C oocyte clamp (Warner Instruments, LLC, Hamden, CT, USA). Oocytes were placed in a RC-3Z oocyte recording chamber (Warner Instruments, LLC) and exposed for 8 s to different concentrations of indole (CAS 120-72-9; Sigma-Aldrich, St. Louis, Mo, USA), (*R*)-1-octen-3-ol (CAS 3687-48-7, 98.2%; Bedoukian Research Inc., Danbury, CT, USA),* N*,*N*-diethyl-m-toluamide (DEET; CAS number 134-62-3; Sigma-Aldrich) and the insect repellent ethyl butylacetylaminopropionate (IR3535; CAS 52304-36-6; Merck & Co., Inc., Kenilworth, NJ, USA). All compounds were solubilized in 200 μl of dimethyl sulfoxide (DMSO) prior to dilutions in Ringer’s buffer. *Orco* alone was expressed in oocytes and exposed to *N*-(4-ethylphenyl)-2-{[4-ethyl-5-(pyridin-3-yl)-4*H*-1,2,4-triazol-3-yl]sulfanyl}acetamide (VUAA1; Innovapharm Ltd., Royal Sutton Coldfield, UK) as a positive control. Currents were allowed to return to baseline between odorant applications. Data acquisition were carried out with a Digidata 1550A and pCLAMP10 (Molecular Devices, Sunnyvale, CA, USA). Plotting and statistical analyses of the data were performed using GraphPad Prism 7 (GraphPad Software Inc., La Jolla, CA, USA). To determine the sensitivity of OR8 towards the different indole treatments, we interpolated the half-maximal effective concentration (EC_50_) values, which correspond to the ligand concentrations eliciting 50% of the maximal current response.

### Arm-in-cage assay for human host-seeking behavior

To investigate the potential role of indole in human host-seeking behavior, we developed an arm-in-cage bioassay in which the experimenter’s hand is presented to fifteen 5–10-day-old post emergence adult females. The bioassay comprises custom-designed three-dimensional-printed interlocking ring that creates a 55-mm-diameter opening in a powder-free latex glove worn by the experimenter. A plastic screen is wedged between the two ring components to protect against mosquito bites. The ring supports an odorant delivery platform located at the center of the ring, which receives a 10-mm-diameter cover glass and two 5-mm-diameter filter discs (WHA10016508; Merck) (Additional file 1: Figure S1, Additional file 2, Additional file 3). Mosquitoes are introduced in a 20.3-cm^3^ metal cage located inside an incubator for at least 10 min before each experiment (26 °C, 80% RH) for acclimatation. This cage was chosen based on an earlier study reporting the effects of repellents on flight approach in *Ae. aegypti* [2]. The cage is then placed at room temperature (23–25 °C) under a video camera (EOS 70D, lance: MACRO 0.25/0.8ft; Canon Inc., Tokyo, Japan) to monitor and quantify the numbers of mosquito visits and their duration on the plastic screen. We used the solvent diethyl ether (DEE) as a vehicle. On a blank filter disc, 25 μl of DEE, indole or DEET was deposited on the filter paper and allowed to evaporate for 2 min prior to mosquito exposure. We used tenfold dilutions of indole ranging from 10^–6^ to 10^–1^ M, and 10^–1^ M DEET. The experimenter rubbed the ring-mounted glove against the shirt and skin. All experiments were conducted during the first 3 h of the diurnal period and lasted 10 min. This schedule was chosen for practical reasons and also because mosquitoes consistently exhibited attraction to the human hand. To determine the repellency of indole against human host-seeking mosquitoes, we recorded the number of females landing per minute and counted the duration of their stay on the screen surface. Mosquito landing count was normalized (${x}^{^{\prime}}= \frac{x-\mathrm{min}(x)}{\mathrm{max}\left(x\right)-\mathrm{min}(x)}*100)$. Mosquitoes returning to the region of interest were counted as a new visit. Statistical comparisons of mosquito landing were carried out with a Kruskal–Wallis H-test and post-hoc pairwise comparisons were carried out using the Wilcoxon rank sum exact test (*P*-value adjustment method: Benjamini-Hochberg [BH]). A one-way analysis of variance (ANOVA) analysis was used to analyze mosquito duration.

### Wind tunnel bioassay

A wind tunnel system was used to measure the response of female *Ae. aegypti* to CO_2_ alone, 1-octen-3-ol and indole stimuli. The wind tunnel is a rectangularly shaped chamber (2200 × 500 × 500 mm) composed of three compartments: a release, flight and odor delivery chamber, respectively. The release chamber holds the mosquitoes prior to the experiment, while the odorant chamber releases an odor from a clean air delivery system (Sigma Scientific LLC., Micanopy, FL, USA). Ten females (same age as previously described) were transferred to a release box (200 × 160 × 200 mm) inside the release chamber (500 × 200 × 500 mm) and allowed to acclimate for 1 h in darkness at 27 ± 0.7 °C. Experiments were conducted between 10:00 and 13:00 local time. Three experiments were conducted for each treatment. The flight tunnel (1500 × 500 × 500 mm) was illuminated with five infra-red lights (RT VAR2-i2-1; Raytec Ltd., Ashington, UK), and a laminar air flow was applied at a speed of 0.1 m/s. After acclimatization, the injection air flow was manually opened for 15 min (duration of each experiment) with compressed air (4 l/m), which delivered the stimulus. Immediately afterwards, flight coordinates were recorded using TrackIt system (SciTrackS GmbH, Pfaffhausen, Switzerland) and the release box door opened. The time interval between each experiment was at least 30 min under constant room ventilation with the wind tunnel top cover removed. Manual maintenance of the wind tunnel was carried out using nitril latex-free gloves to prevent odor contamination.

### Tracking software and data analysis

Flight trajectories were recorded using two acA2000-165um infrared-sensitive cameras (Basler AG, Germany) and processed by the 3D software TrackIt (SciTrackS GmbH, Switzerland). Output data was analyzed through R version 4.0.2 programing language [24]. A description of the functions to quantify parameters of flight trajectories can be found in Additional file 4: Table S1. In total, the combined treatments consisted of 90m614 raw *X*, *Y*, *Z* coordinates. Coordinates located outside the flight tunnel (reflection) were removed. The visual field was cropped to define a 800 × 500 × 500 region of interest (ROI; Fig. 5a). Coordinates within the first 3 min of recording were retained and analyzed. We randomly sampled 25% of the remaining coordinates (5192 coordinates) for statistical analysis. Statistical comparisons between treatments were carried out with a Kruskal–Wallis H-test, and post-hoc pairwise comparisons were carried out using the Wilcoxon rank sum exact test (*P* value adjustment method: Bonferroni).

### Odorant stimuli

The injected stimuli included: (i) CO_2_ (600 ± 20 ppm) + 25 µl of DEE; (ii) CO_2_ + 25 µl of DEE + 2 µl of 1-octen-3-ol (168 mg); and (iii) CO_2_ + 25 µl of DEE + 2 µl of 1-octen-3-ol (168 mg) with indole 5 M (14.625 mg). Each stimulus was deposited on a double Whatman filter paper (WHA10016508; Merck & Co., Inc.) as described above in the arm-in-cage bioassay. To allow DEE to evaporate, the holder was placed for 2 min in a separate room, following which the chemical holder was placed inside an OSI-4550 inline volatile collection chamber (Sigma Scientific LLC) connected between the clean air delivery system and the injector. CO_2_ was delivered by a steel compressed gas cylinder, and its content was monitored at the injector site using a TES-1370 CO_2_ analyser (TES Electrical Electronic Corp., Taipei, Taiwan). During all these procedures, the person conducting the experiment wore laboratory gloves and a mask.

## Results

### Indole inhibits AaegOR8 activation by (*R*)-1-octen-3-ol

Based on the chemical similarities between indole and DEET (Fig. 1a), we surmised that indole would exert an inhibitory effect on AaegOR8. A 10^–3^ M indole concentration reduced by approximately 30% the AaegOR8 current amplitude elicited by 10^–7^ M (*R*)-1-octen-3-ol (Fig. 1b). This effect was not observed in *Orco*-injected oocytes.

To determine the nature of this inhibitory effect, we established a series of concentration–response curves using OR8-Orco-injected oocytes exposed to increasing concentrations of (*R*)-1-octen-3-ol alone or combined with 10^–4^ M, 10^–3^ M or 10^–2^ M indole (Fig. 1c). Two types of currents were observed, including the expected agonist-induced depolarization currents and unusual indole-dependent hyperpolarization or reduction in baseline currents.

The interpolated EC_50_ values for octenol alone versus 10^–4^ M and 10^–3^ M indole were not statistically significant (Fig. 1d; Additional file 5: Table S2). The EC_50_ value elicited by 10^–2^ M indole was significantly different from that elicited by (*R*)-1-octen-3-ol alone, but the response was moderate. These findings suggest that indole does not have an important effect on the sensitivity of this receptor for (*R*)-1-octen-3-ol. Contrary to our initial results (Fig. 1b), we did not observe any significant inhibition of the amplitude response with increasing indole concentration (Fig. 1e). However, current amplitudes are contingent on oocyte inherent variability. To address this limitation, we determined the systematic effect of indole on depolarization current amplitudes by normalizing all of the current responses to the initial 10^–7^ M (*R*)-1-octen-3-ol exposure (Fig. 1c). We noted that 10^–2^ M indole consistently reduced the current amplitude across the concentration of (*R*)-1-octen-3-ol, excluding 10^–10^ M and confirmed that 10^–3^ M indole significantly reduced the response amplitude of AaegOR8 to 10^–7^ M (*R*)-1-octen-3-ol (Fig. 1f).

**Fig. 2:**
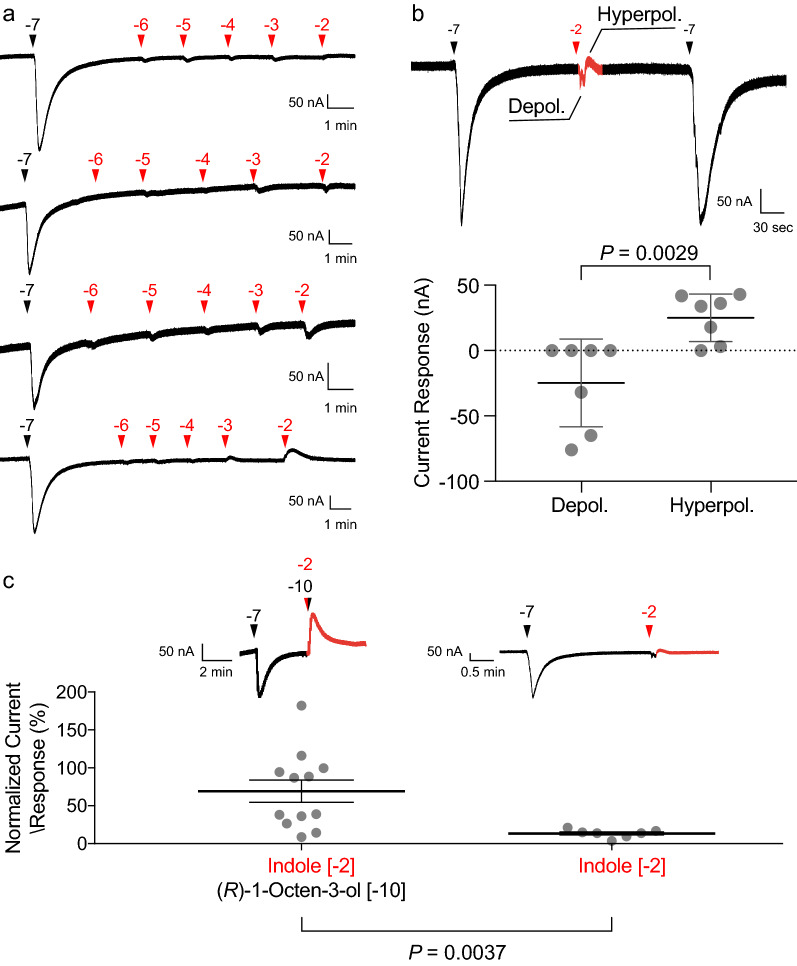
High indole concentrations elicit both depolarization and hyperpolarization currents. **a** Representative current traces of AaegOR8-Orco activation by 8-s-long stimulations of (*R*)-1-octen-3-ol alone or increasing indole concentrations (10^–6^–10^–2^ M). Arrowheads above the traces indicate the onset of the odorant stimulus, black arrowheads represent (*R*)-1-octen-3-ol and red arrowheads represent indole. **b** Representative current trace of AaegOR8-Orco activation by 10^–7^ M (*R*)-1-octen-3-ol (black arrowhead) and by 10^–2^ M indole (red arrowhead). Statistical significance was determined using the Wilcoxon–Mann–Whitney test (*n* = 7). **c** (*R*)-1-Octen-3-ol was required to elicit significant indole-induced hyperpolarization currents. Nanomolar concentrations of (*R*)-1-octen-3-ol induced larger indole-induced depolarization currents than indole alone. Representative traces are shown above. Statistical significance was determined using a *t*-test (*n* = 7–12)

Indole concentrations of 10^–3^ M and 10^–2^ M elicited hyperpolarization currents (reductions in current baseline) in the presence of (*R*)-1-octen-3-ol concentrations ranging from 10^–10^ M to 10^–7^ M (Fig. 1c). These hyperpolarization currents were concentration dependent and were surmounted at higher (*R*)-1-octen-3-ol concentrations (Fig. 1g). To understand the contribution of indole alone to these currents, we exposed OR8 to increasing concentrations of indole without any other ligand present (Fig. 2a) and observed that 10^–6^ to 10^–4^ M indole evoked small depolarization currents. The two highest indole concentrations elicited either depolarization or hyperpolarization currents that were a fraction of those elicited by the initial 10^–7^ M (*R*)-1-octen-3-ol stimulation. To better characterize these small yet inconsistent effects, we focused on the currents elicited by 10^–2^ M indole and consistently observed these small hyperpolarization and hyperpolarization currents (Fig. 2b). By comparison, this same concentration of indole, in the presence of (*R*)-1-octen-3-ol at concentrations as low as 10^–10^ M, elicited currents reaching the initial response to 10^–7^ M (*R*)-1-octen-3-ol, suggesting that the significant indole-induced hyperpolarization currents require the presence of (*R*)-1-octen-3-ol (Fig. 2c).

### Indole modifies the OR8-mediated current baseline of the oocyte membrane.

**Fig. 3:**
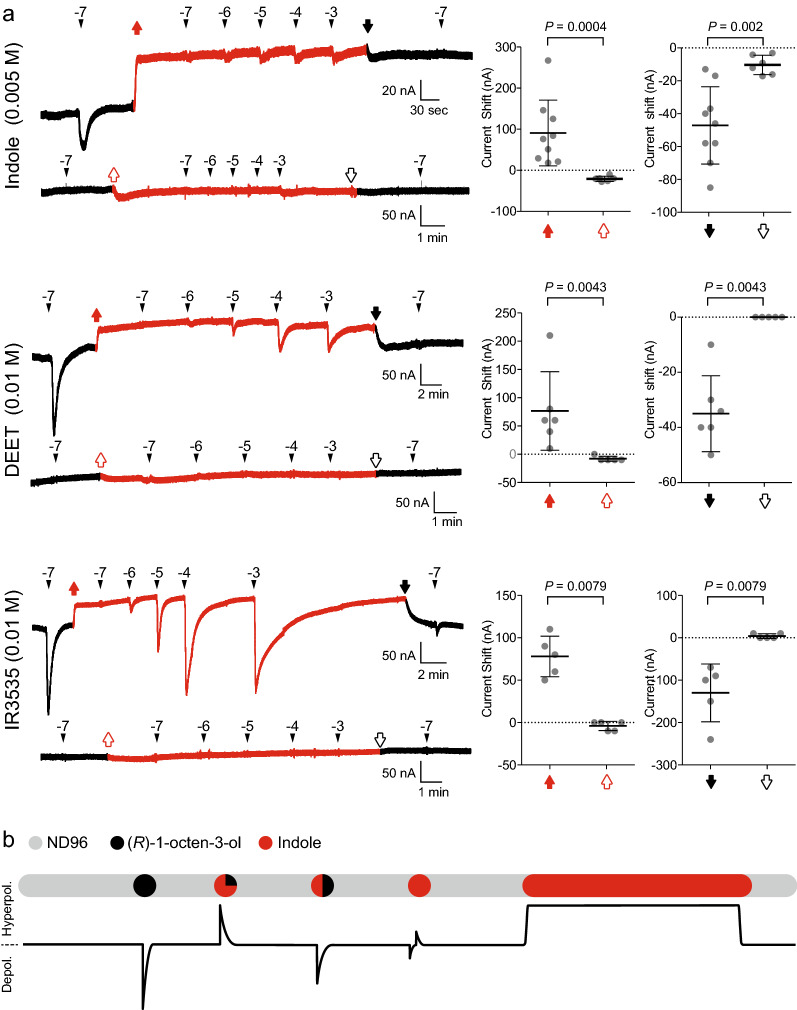
Indole and insect repellents (DEET, IR3535) increased the baseline current state of AaegOR8-Orco-injected oocytes. **a** Representative current traces of AaegOR8-Orco successively exposed to 100 nM (*R*)-1-octen-3-ol in ND96 buffer (saline; black trace) and continuous perfusion under indole (0.005 M), DEET (0.01 M) and IR3535 (0.01 M) perfusions (red traces), with increasing concentrations of (*R*)-1-octen-3-ol, ND96 buffer perfusion and 100 nM (*R*)-1-octen-3-ol. The same protocol was also applied to water-injected oocytes. In AaegOR8-Orco-injected oocytes, perfusion buffer transitions from ND96 buffer to indole, DEET or IR3535 are shown as solid red arrows, while the transitions in the reverse direction are shown as a solid black arrow. In water-injected oocytes, the corresponding buffer transitions are indicated by empty red and black arrows. The baseline currents of OR8-Orco-injected oocytes exhibited significant increases and decreases compared to those of water-injected oocytes. Statistical significance was determined using the Wilcoxon–Mann–Whitney test (*n* = 5–9). **b** Under saline perfusion (ND96), AaegOR8-Orco elicitd a stable current baseline. A stimulus pulse of (*R*)-1-octen-3-ol activated AaegOR8-Orco, which translated into a transient whole-cell depolarization current. A pulse of (*R*)-1-octen-3-ol (low concentration) and indole (high concentration) mixture transiently induced a hyperpolarization current. Increasing (*R*)-1-octen-3-ol concentrations reversed indole blockade. High indole concentrations elicited small depolarization and hyperpolarization currents. Under indole perfusion, the current baseline was increased. IR3535, Ethyl butylacetylaminopropionate

The cause of the observed hyperpolarization currents caused by indole in the presence of (*R*)-1-octen-3-ol required further investigation. It mirrored a phenomenon previously documented with AaegOR8, AaegOR2 and AaegOR10 [5, 7] and more recently with additional ORs from *Cx. quinquefasciatus*, *Ae. aegypti*, and *An. gambiae* [25]. To investigate whether the hyperpolarization current was a transient response or a durable modification of the current baseline, we exposed OR8-injected oocytes to a change of perfusion buffer by switching from the ND96 buffer solution to a 5.10^–3^ M indole perfusion. We also administered increasing 10-fold dilutions of (*R*)-1-octen-3-ol before reversing the perfusion solution back to ND96 (Fig. 3a). Prior to and after the exchange of the two perfusion buffers, the oocyte was exposed to a transient stimulation of 10^–7^ M (*R*)-1-octen-3-ol for control purposes. The switch from ND96 buffer to indole elicited a stable decrease in the baseline current not observed in water-injected oocytes (Fig. 3a). (*R*)-1-Octen-3-ol produced very little depolarization current at all tested concentrations. By comparison, DEET and IR3535 evoked larger currents. The switch in buffers in the opposite direction exhibited a stable decrease in baseline current that was most pronounced in the case of DEET and IR3535 (Fig. 3a). In these experiments, we treated the oocytes with a lower indole concentration, as compared to DEET and IR3535, because 10^–2^ M indole consistently killed the perfused oocytes. All observed currents elicited by (*R*)-1-octen-3-ol, indole, a mixture of these two ligands and buffer switch are summarized in Fig. 3b.

### Indole inhibits close-range human-host attraction.

**Fig. 4:**
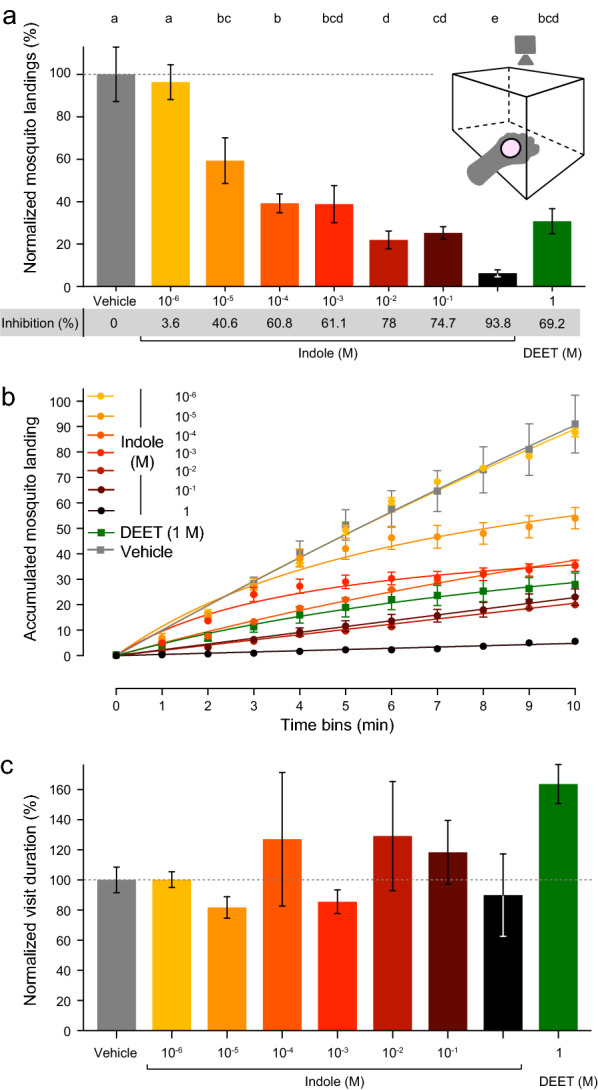
Indole repels human-seeking female *Aedes aegypti* mosquitoes. **a** Diagram of the arm-in-cage assay (inset). The gloved had of the person participating in the experiment is introduced inside a cage containing 15 *Ae. aegypti* female mosquitoes. The exposed skin area was monitored by a recording camera (see Additional file 6: Video S1 for a representative video). The number of mosquito visits were counted and normalized as a function of treatments, including treatments with the vehicle (diethyl ether [DEE]), DEET 1 M and 7 indole concentrations. All treatment groups were analyzed using a Wilcoxon rank-sum test (*n* = 3, see Methods section for more details). The percentage of inhibition is indicated below the graph in the gray shaded area. Different lowercase letters above bars indicate significant difference at *P* ≤ 0.05). **b** Hyperbola line fitting of the cumulative number of mosquito landings over time. Points represented are mean ± standard deviation of the mean (*n* = 3). See Additional file 7: Fig. S2. **c** Histogram of the normalized average time duration mosquitoes spent on the exposed skin area. A one-way ANOVA analysis was used to analyze all treatment groups. The Shapiro–Wilk normality test (*W* = 0.95056, *P* = 0.2212) was performed on the square root of the dependent variable (*n* = 3). Vertical lines indicate SEM

To explore the behavioral role of indole, we exposed a human hand to female mosquitoes using an arm-in-a-cage assay (Fig. 4a insert). The hand was covered with a protective glove which allowed mosquitoes to detect human skin odor through a window created by an open area on the dorsal side of the hand (Additional file 6: Video S1). This open area was protected by a screen and was equipped with an odor delivery system (Additional file 1: Figure S1, Additional file 2, Additional file 3). Increasing doses of indole ranging from 10^–6^ to 10^–1^ M were deposited on this delivery system, and repellency was measured in terms of number of mosquito visits and duration of visits. The repellency effect of DEET was significantly higher than that of the DEE vehicle and indole 10^–6^ M, respectively (Fig. 4a) (Kruskal–Wallis H-test, *H* = 106.11, *df* = 8, *P* < 0.0001). All treatments with indole, except for 10^–6^ M, were significantly different those with the vehicle (Fig. 4a). Increasing indole doses reduced the number of mosquito visits from 40.6% to 93.8%. We observed a 3.6% inhibition with a 10^–6^ M indole, but this effect was not statistically significant. Looking at the accumulated landing numbers, DEE vehicle and 10^–6^ M indole elicited overlapping temporal dynamics (Fig. 4b). Indole at 10^–1^ M had a significantly higher temporal repellency than all other treatments, including DEET at the same concentration. Other indole treatments exhibited intermediate temporal repellency between these two extremes. However, the only significant differences were observed between the vehicle and 1 M indole (Additional file 7: Fig. S2). In terms of visit durations, mosquitoes spent on average the same amount of time on the open area when landing occurred (Fig. 4c) (ANOVA, *F*_(8,18)_ = 1.219, *P* = 0.343).

### Indole reduces 1-octen-3-ol-mediated attraction.

**Fig. 5:**
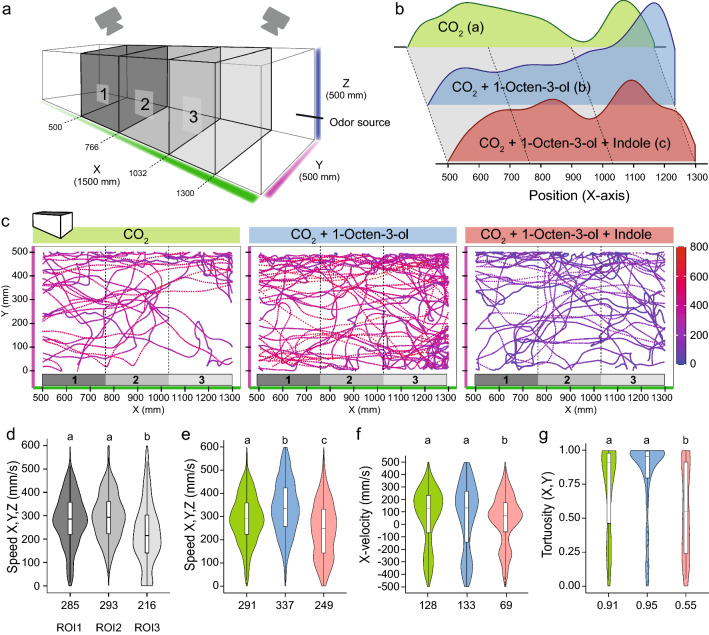
Indole reduces anemotactic behavior induced by CO_2_ and 1-octen-3-ol. **a** Schematic of the flight tunnel. The ROI was equally divided into three sections represented by differentially shaded volumes (ROI-1, -2, -3). **b** Kernel density estimations of mosquito coordinates within the first 3 min of odorant-induced flight in ROI-1, -2 and -3. Statistical significance is given in parentheses (*P* ≤ 0.05). **c** Bird’s-eye view of the collective flight trajectories elicited by the three odor treatments. Each visible dot represents a trajectory coordinate. Speed levels along the trajectory are color-coded according to the speed index (mm/s). Graded areas below the* X*-axes represent the three sections of the ROI (1, 2, 3). **d** Violin plot of mosquito speed in ROI-1, -2 and -3 in response to CO_2_. **e** Violin plot of mosquito speed in ROI-1 and -2 as a function of the odor treatments. **f** Violin plot of upwind velocity in ROI-1 and - as a function of the odor treatments. **g** Violin plot of tortuosity in ROI-1 and -2 as a function of the odor treatments. In all violin graphs (**e**–**g**), the CO_2_ treatment is colored green, the CO_2_ + 1-often-3-ol treatment is colored blue and the CO_2_ + 1-octen-3-ol + indole treatment is colored maroon. CO_2_ concentration: 600 ppm; 1-octen-3-ol concentration: 168 mg; indole concentration: 14.625 mg. ROI, Region Of Interest. Different lowercase letters above all violin graphs indicate significant difference at *P* ≤ 0.05). Median values (speed, velocity and tortuosity) are provided below each violin graph

The results of the present study up to this point provided circumstantial pharmacological evidence that indole may in part affect OR8-mediated detection, but they did not give any direct indication that indole affects 1-octen-3-ol-mediated attraction in the context of human host-seeking. To explore this possibility, we used a flight tunnel (Fig. 5a) to expose human-host seeking female mosquitoes to a synthetic blend composed of CO_2_ and 1-octen-3-ol. We used three odor treatments, including CO_2_ alone or in combination with 1-octen-3-ol and indole, respectively. The ROI was divided into three sections (ROI-1, 2, 3; Fig. 5a) to explore possible differences in terms of trajectory speed, velocity and tortuosity. Kernel density estimations of mosquito locations along the *X*-axis were statistically different between the three treatments (Fig. 5b). A bird’s-eye view (*X*–*Y* plane) of flight trajectories representing flight speed suggested differences between the treatments (Fig. 5c; see example of individual trajectories in Additional file 8: Fig. S3). 1-Octen-3-ol seemed to increase the number of trajectories and coverage of the ROI while indole appeared to reduce flight speed across that same area. Among all three sections of the ROI, speed was higher and more consistent in response to CO_2_ in ROI-1 and -2 than in ROI-3 (Fig. 5d) (Kruskal–Wallis H-test, *H* = 86.8, *df* = 2, *P* < 0.0001). As a result, we focused on ROI-1 and -2 for further analyses. As reflected in Fig. 5c, higher speeds were elicited with the addition of 1-octen-3-ol than with CO_2_ alone (Fig. 5e). The addition of indole elicited significant decreases in speed compared to those observed with CO_2_ alone or those in combination with 1-octen-3-ol (Kruskal–Wallis H-test, *H* = 301.2, *df* = 2, *P* < 0.0001). While upwind velocity did not show any statistical differences between CO_2_ and 1-octen-3-ol, indole elicited lower upwind velocities (Fig. 5f) (Kruskal–Wallis H-test, *H* = 35.5, *df* = 2, *P *< 0.0001). Finally, indole-induced tortuosity was significantly higher than the tortuosity elicited by CO_2_ alone or in combination with 1-octen-3-ol (Fig. 5g) (Kruskal–Wallis H-test, *H* = 20.9, *df* = 2, *P* < 0.0001).

## Discussion

The overlapping chemical structures between DEET and indole provided the initial impetus to test the potential blocking effect of the latter on AaegOR8. We observed a significant reduction in the current baseline of OR8 at high indole concentrations, with indole generating (*R*)-1-octen-3-ol-dependent hyperpolarization currents (Fig. 1c). In comparison to DEET and IR3535, these increases in current baseline were robust and exhibited different degrees of reversibility, with indole showing the highest level of insurmountability (i.e. 1-octen-3-ol failed to elicit significant responses and the switch to ND96 biffer did not restore the baseline current). This finding indicates that the affinity of indole for OR8 is higher than that of the other two tested insect repellents. We also did not observe that indole affected whole-cell currents of oocytes expressing Orco, suggesting that indole acts as an allosteric modulator of OR8.

We had previously reported on these currents, elicited by the highest concentrations of DEET, IR3535 and picaridin, without providing a molecular mechanism underlying this phenomenon [5, 7]. Recently, similar observations were reported with *Cx. quinquefasciatus* and *An. gambiae* ORs, suggesting that these hyperpolarization currents may be mediated by chloride influx [25, 26]. The reversible decrease of the baseline current in OR8-injected oocytes suggests that these hyperpolarization currents may not be generated by the activation and subsequent opening of the OR8 ion channel but, rather, may reflect a reduction of its constitutive activity. This interpretation is consistent with the observed moderate yet significant increase in baseline current.

What is the ecological role of (*R*)-1-octen-3-ol? *OR8* is expressed in the maxillary palps of adult mosquitoes [11, 12]. It is selectively activated by nanomolar concentrations of (*R*)-1-octen-3-ol when expressed in *Xenopus* oocytes [13]. The evidence presented in this study suggests a correlative relationship between the observed repellency and one possible molecular mechanism for eliciting this behavior. We recognize that indole repellency may be generated by other molecular targets, such as the indolergic or other receptors. Indeed, indole-induced repellency may have no direct relevance to 1-octen-3-ol/OR8-mediated behavior. The high concentrations used in the present study (up to 1 M) are unlikely to be found in nature. However, at the lower used concentrations, we also observed significant repellency. Whether the same or other detection mechanisms are involved is not known. It is possible that different indole concentrations target different molecular mechanisms, as has been observed for DEET [5, 6, 27–31].

Indole is synthesized by bacteria [32, 33], fungi and plants [34] and released by animals [35] and may attract female mosquitoes in the contexts of animal host- [36] and oviposition-seeking [37]. In *Culex* mosquitoes, indole has been associated with fermented Bermuda grass infusions, which attract gravid female mosquitoes [38–40]. However, indole alone does not appear to act as an oviposition attractant in *Ae. aegypti* [19] or in *An. gambiae*, while it is slightly repellent to ovipositing *Aedes albopictus* [19].

Indole may play a role in human host-seeking behavior as well [37]. Microbiota on the skin [41] and in sweat [35] release indole and may contribute to its attractiveness. We tested the olfactory-mediated effect of multiple indole doses in the context of human host-seeking behavior and provide observational evidence that indole repels female mosquitoes (Fig. 4). Whether these doses are ecologically relevant is not known, but this observation could provide one explanation as to why different individuals exhibit different levels of attractiveness towards mosquitoes.

To test whether indole-mediated OR8 inhibition would elicit repellency, we tested its effect against blood-seeking female *Ae. aegypti*. Our results suggest that indole acts as an olfactory repellent in a broad range of doses (1 M–10^–5^ M) and loses its repellent activity at low doses (10^–6^ M). We used DEET as a positive control since it is a recognized and effective insect repellent. DEET probably operates through different modes of action, including interacting with both smell and taste receptors [5, 6, 27–31] and by reducing the volatility of odorants [9]. In our arm-in-a-cage assay, indole was not applied to the skin but rather onto a physically separate chemical holder, suggesting that the inhibitory effect of indole is not that of chemically decreasing odorant volatility (‘masking’), as has been suggested in *An. gambiae* [8–10]. The number of mosquito visits was the most significant measure of repellency. By contrast, the durations of the visits were highly variable, with DEET standing out as compared to the vehicle. However, all of the tested treatments did not show any significant differences. Taken together, these results suggest that mosquitoes were repelled before making contact with the screen, which is located immediately below the chemical source. This would be consistent with an olfactory-mediated effect, whereas once landed on the screen, olfactory information may be downplayed by the brain while other senses, such as taste or close-range chemosensation, take precedence.

The highest indole concentrations used in our behavioral experiments are unpleasant (moth ball) to the human nose and may elicit rejection from consumers. However, we have shown that indole concentrations as low as 10^–5^ M elicit significant repellency at short range. These concentrations are consistent with those available in the fragrance industry, which uses indoles in dilutions of ≤ 0.1% to create a floral effect in perfumes. Overall, indole demonstrates minimal toxicity at low concentrations [42]. When ingested, indole exhibits an LD_50_ of 1000 mg [43]. The quantity we used in our flight tunnel was 14.625 mg, while the minimal dose to repel mosquitoes in our cage assay was 146 µg (10^−5^ M). These quantities are well below the toxicity levels, hence the use of indole in perfumery. In addition, indole may be mixed with other volatiles to enhance this floral effect. While it is not known that perfumes containing indoles repel mosquitoes, other personal care products with plant odorants as part of their formulations have been shown to repel mosquitoes [44, 45]. Future studies on indolic perfumes might also reveal an ability to create a repelling effect.

## Conclusions

The results of our study provide strong support that indole, a mosquito kairomone of unclear ecological significance, inhibits human host-seeking *Ae. aegypti* females. We also provided pharmacological evidence that the OR8/1-octen-3-ol detection pathway is a potential molecular mode of action for this inhibition. Since indole activates multiple mosquito ORs, including OR2 and OR10, the indole-mediated reduction in anemotactic flight may be caused by distinct olfactory pathways. Our observations nevertheless raise the need for additional studies on the efficacy of indole as a potential mosquito spatial repellent.

## Supplementary Information

**Additional file 1: Figure S1.** Diagrams of the odor delivery system.** A** The odor delivery system is composed of interlocking top and bottom rings attached to a removable chemical holder (overview, top, bottom and side views are provided). Dimensions are provided in millimeters.** B** The rings are locked in place across the glove of the person conducting the experiment. The glove within the inner area of the ring is removed, exposing the skin surface. A plastic net is intercalated between the rings and serves to physically protect the skin from mosquito bites.

**Additional file 2: **Hand rings.stl. File format for 3D-printing of the two complementary hand rings.

**Additional file 3: **Chemical holder.stl. File format for 3D-printing of the chemical holder component.

**Additional file 4: Table S1.** Scripts and functions used to calculate flight parameters.

**Additional file 5: Table S2.** EC_50_ values (Log [M]) of AaegOR8-Orco concentration-response relationships.

**Additional file 6: Video S1.** Representative videos of arm-in-cage assays. Side-by-side recorded sessions of the first 5 min of a control experiment (vehicle) and an indole (0.1 M) treatment

**Additional file 7: Figure S2.** High indole concentrations exhibit significant repellency over time. Cumulative number of mosquito landings per elapsed minute (see Fig. 4b). Points represented are mean ± SEM (*n* = 3). Statistical significance was determined using a non-parametric test followed by a Dunn’s multiple comparisons test (*P* values shown on the histograms).

**Additional file 8: Figure S3.** Representative single flight trajectories of female mosquitoes exposed to CO_2_, 1-octen-3-ol and indole, respectively.** A** Schematic of the flight tunnel and the overall region of interest (ROI) located between 500 and 1300 mm on the* X*-axis (shaded gray box). The three odor treatments are color-coded.** B** Example trajectories of mosquitoes exposed to CO_2_ or to a combination of CO_2_ + 1-octen-3-ol, or CO_2_ + 1-octen-3-ol + indole. These trajectories were recorded in the ROI within the flight tunnel and projected into each of the three 2-dimensional planes (from left to right, Y-X, Z-X, and Z-Y). Speed is color-coded according to the speed index. The last coordinate of the trajectory is shown in black and marked with an arrow. The dashed line indicates trajectory segments outside the ROI.

## Data Availability

Behavior assays and electrophysiology results underlying this article will be shared on reasonable request to the corresponding author.
