# Supplementary figures and images for: The ovipositor cue indole inhibits animal host attraction in Aedes aegypti (Diptera: Culicidae) mosquitoes
Source: Parasit Vectors. 2022 Nov 12;15:422. doi: 10.1186/s13071-022-05545-8 (PMC9652956; doi:10.1186/s13071-022-05545-8)

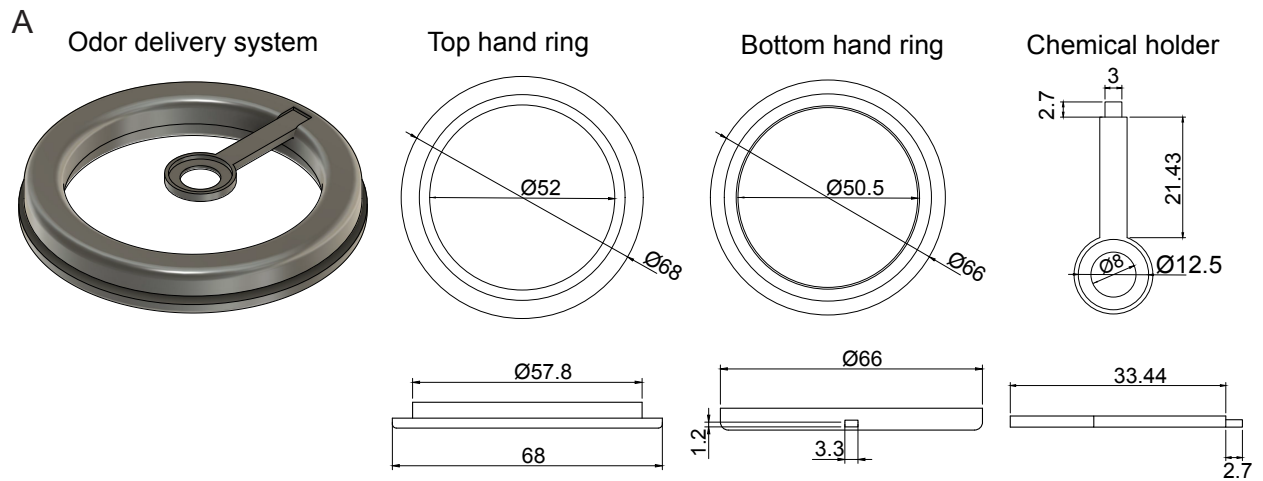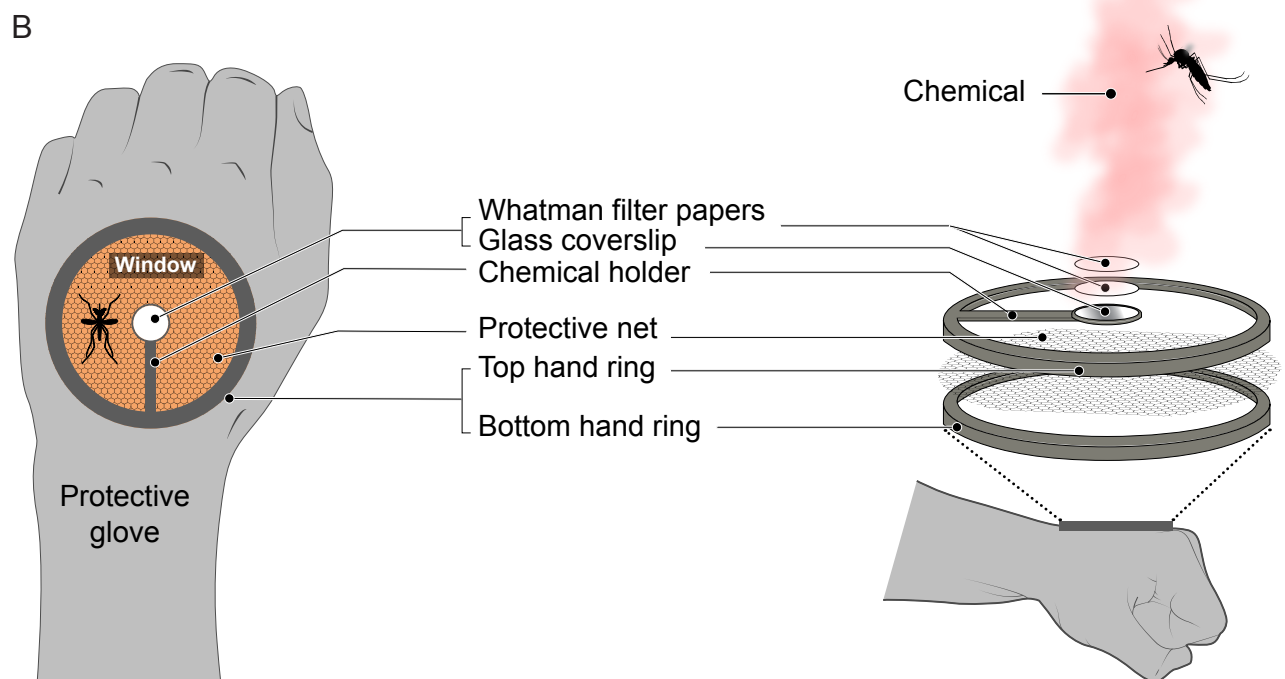

Supplement: Supplementary file 1 — Additional file 1: Figure S1. Diagrams of the odor delivery system. A The odor delivery system is composed of interlocking top and bottom rings attached to a removable chemical holder (overview, top, bottom and side views are provided). Dimensions are provided in millimeters. B The rings are locked in place across the glove of the person conducting the experiment. The glove within the inner area of the ring is removed, exposing the skin surface. A plastic net is intercalated between the rings and serves to physically protect the skin from mosquito bites. [file 13071_2022_5545_MOESM1_ESM.pdf]

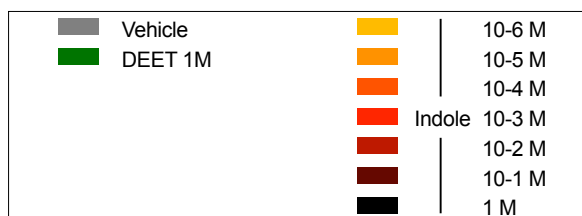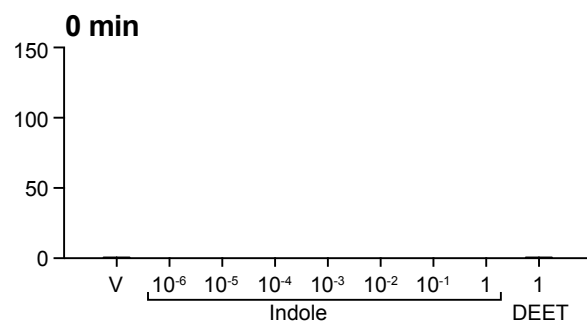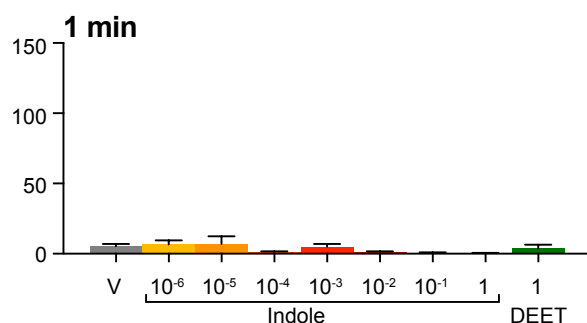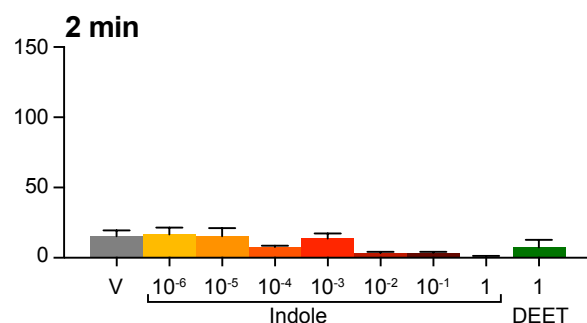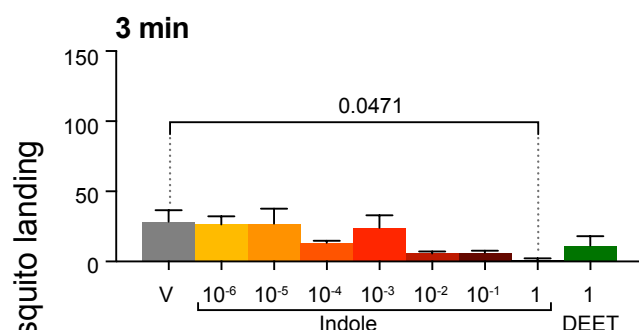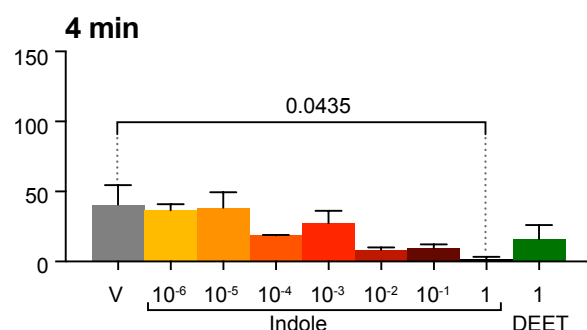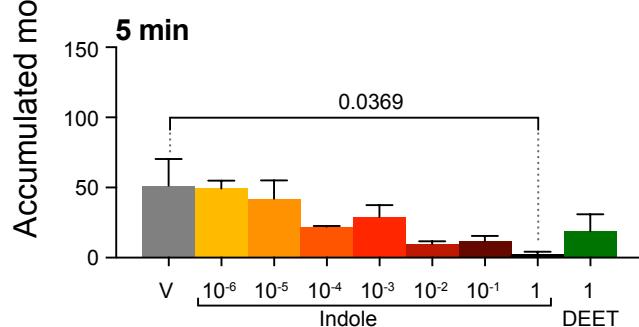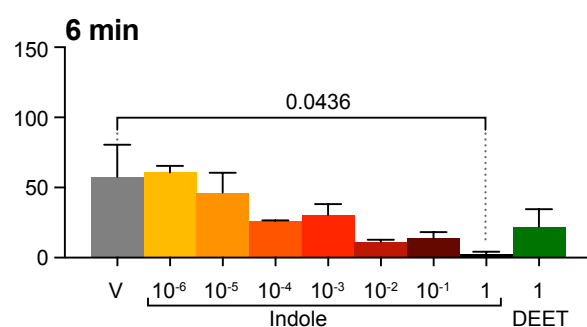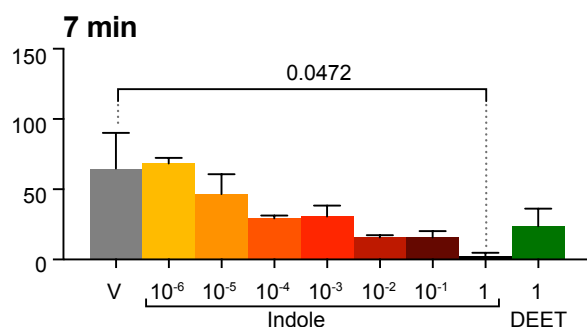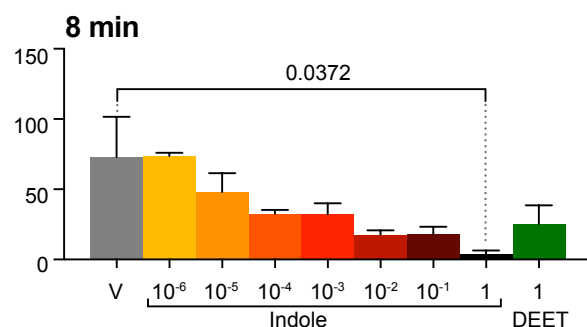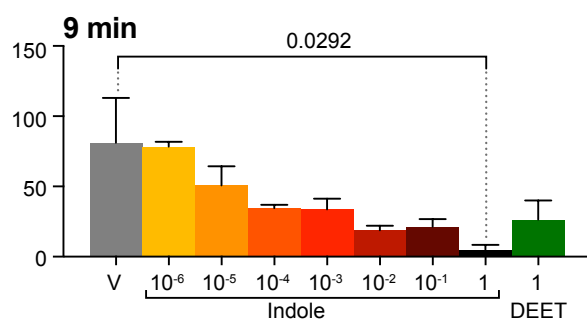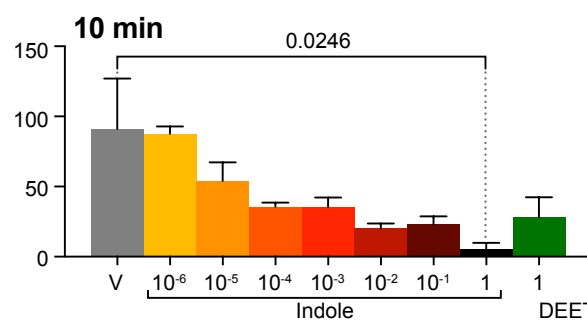

Supplement: Supplementary file 7 — Additional file 7: Figure S2. High indole concentrations exhibit significant repellency over time. Cumulative number of mosquito landings per elapsed minute (see Fig. 4b). Points represented are mean ± SEM (n = 3). Statistical significance was determined using a non-parametric test followed by a Dunn’s multiple comparisons test (P values shown on the histograms). [file 13071_2022_5545_MOESM7_ESM.pdf]

A

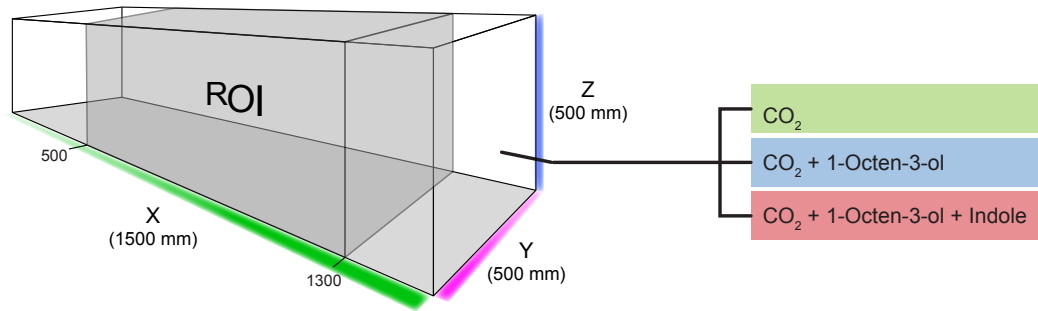

B

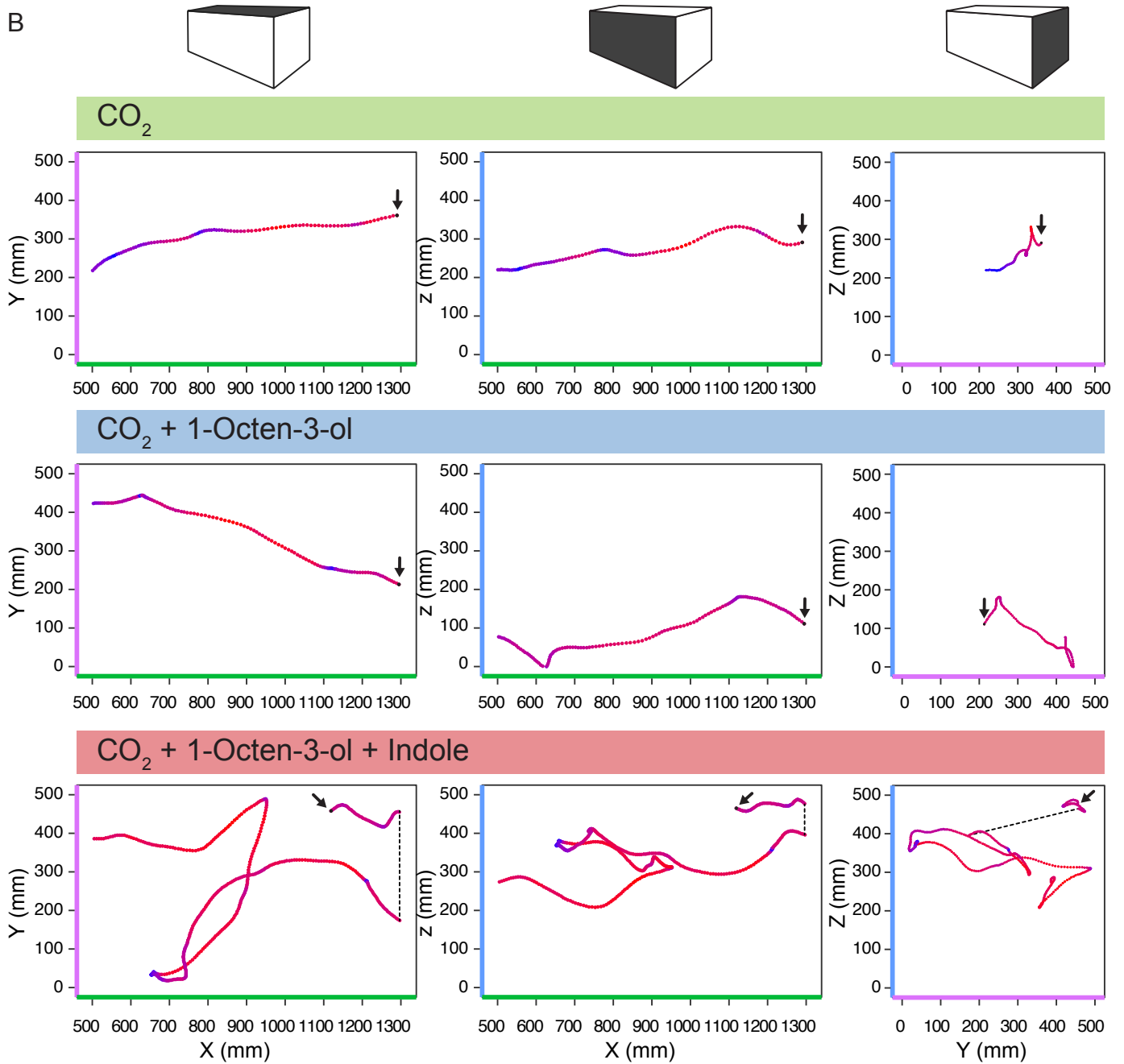

Speed index  
(mm/sec)

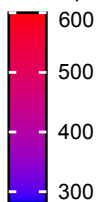

Supplement: Supplementary file 8 — Additional file 8: Figure S3. Representative single flight trajectories of female mosquitoes exposed to CO2, 1-octen-3-ol and indole, respectively. A Schematic of the flight tunnel and the overall region of interest (ROI) located between 500 and 1300 mm on the X-axis (shaded gray box). The three odor treatments are color-coded. B Example trajectories of mosquitoes exposed to CO2 or to a combination of CO2 + 1-octen-3-ol, or CO2 + 1-octen-3-ol + indole. These trajectories were recorded in the ROI within the flight tunnel and projected into each of the three 2-dimensional planes (from left to right, Y-X, Z-X, and Z-Y). Speed is color-coded according to the speed index. The last coordinate of the trajectory is shown in black and marked with an arrow. The dashed line indicates trajectory segments outside the ROI. [file 13071_2022_5545_MOESM8_ESM.pdf]
