# Supplementary material for: The ovipositor cue indole inhibits animal host attraction in Aedes aegypti (Diptera: Culicidae) mosquitoes
Source: Parasit Vectors. 2022 Nov 12;15:422. doi: 10.1186/s13071-022-05545-8 (PMC9652956; doi:10.1186/s13071-022-05545-8)
Supplement: Supplementary file 4 — Additional file 4: Table S1. Scripts and functions used to calculate flight parameters. [file 13071_2022_5545_MOESM4_ESM.docx]

**Supplementary table II. Scripts and function used to calculate flight parameters.**

| **Function calculation** | **Calculation method** |
| --- | --- |
| X, Y and Z velocity | TrackIt (SciTrackS) |
| Speed | $v_{i}= \sqrt{\vec{v}_{xi}^{2}+ \vec{v}_{yi}^{2} {+ \vec{v}}_{zi}^{2}}$  $v$ = speed  $\vec{v}$: velocity  $i:$ a given coordinate |
| Tortuosity | $T_{ti}= \frac{C_{ti}}{L_{ti}}$  $ti$ = trajectory number  $C$: length of each trajectory (measured along the x-y axis)  $L:$ distance between first and last trajectory coordinate (measured along the x-y axis) |
| Kernel density estimation, speed index and violin plots | R package “ggplot2” - Create Elegant Data Visualisations Using the Grammar of Graphics |
| Statistics | R package “stats” - The R Stat package |
| Letters representing statistical differences | R package “rcompanion” - Functions to Support Extension Education Program Evaluation R package “multcompView” - Visualizations of Paired Comparisons |
